# “The Wandering Nerve Linking Heart and Mind” – The Complementary Role of Transcutaneous Vagus Nerve Stimulation in Modulating Neuro-Cardiovascular and Cognitive Performance

**DOI:** 10.3389/fnins.2022.897303

**Published:** 2022-06-16

**Authors:** Helena Dolphin, Tim Dukelow, Ciaran Finucane, Sean Commins, Paul McElwaine, Sean P. Kennelly

**Affiliations:** ^1^Department of Age-Related Healthcare, Tallaght University Hospital, Dublin, Ireland; ^2^Department of Medical Gerontology, School of Medicine, Trinity College Dublin, Dublin, Ireland; ^3^Department of Medical Physics, St James’s Hospital, Dublin, Ireland; ^4^Department of Psychology, Maynooth University, Maynooth, Ireland

**Keywords:** vagus nerve stimulation, cognition, neurocardiovascular control, cerebral blood flow, LC-NE system, inhibitory control, executive function

## Abstract

The vagus nerve is the longest nerve in the human body, providing afferent information about visceral sensation, integrity and somatic sensations to the CNS *via* brainstem nuclei to subcortical and cortical structures. Its efferent arm influences GI motility and secretion, cardiac ionotropy, chonotropy and heart rate variability, blood pressure responses, bronchoconstriction and modulates gag and cough responses *via* palatine and pharyngeal innervation. Vagus nerve stimulation has been utilized as a successful treatment for intractable epilepsy and treatment-resistant depression, and new non-invasive transcutaneous (t-VNS) devices offer equivalent therapeutic potential as invasive devices without the surgical risks. t-VNS offers exciting potential as a therapeutic intervention in cognitive decline and aging populations, classically affected by reduced cerebral perfusion by modulating both limbic and frontal cortical structures, regulating cerebral perfusion and improving parasympathetic modulation of the cardiovascular system. In this narrative review we summarize the research to date investigating the cognitive effects of VNS therapy, and its effects on neurocardiovascular stability.

## Introduction

Vagus nerve stimulation (VNS) as a neurostimulation technique and has received renewed attention in recent years. Traditionally invasive VNS (iVNS) devices were sutured under the skin of the chest with a lateral left neck dissection undertaken to expose the left cervical vagus nerve and wrap a stimulating electrode around it. Each iVNS device is costly and up to 30% of patients have side effects post implantation (Morris and Mueller, 1999). Since the development in the early 2000s of peripheral stimulating devices that harness the vagus nerve’s innervation of the skin of the external ear demonstrating efficacy in treating epilepsy, depression and headaches, interest in wider therapeutic potentials of this treatment have grown (Yap et al., 2020).

Declining cognition associated with aging is a burgeoning global health crisis, with at least 152.8 million persons projected to have dementia worldwide by the year 2050 (Nichols et al., 2022). There are few effective treatments for cognitive decline and dementia, with no current cure (Cummings et al., 2021) and although the first disease modifying anti-amyloid agent has been licensed by the FDA (Steinbrook, 2021), more therapies are urgently needed to help alleviate the personal, societal and economic cost of increasing dementia diagnoses (Xu et al., 2017). Impaired cognition is associated with impaired autonomic function, specifically impaired parasympathetic measures of heart rate variability (HRV) (Forte et al., 2019; Cheng et al., 2022; Liu et al., 2022) likely reflective of the complex interplay between cognition and cardiac modulation, *via* the central autonomic network.

Studies of patients with intractable epilepsy and treatment -resistant depression treated with iVNS devices showed signals indicating increased alertness and potentially cognitive improvements (Ghacibeh et al., 2006a; McGlone et al., 2008; Schevernels et al., 2016; Sun et al., 2017; van Bochove et al., 2018) and a small pilot study investigated iVNS devices in patients with Alzheimer’s Disease with overall positive results (Sjögren et al., 2002; Merrill et al., 2006). Recent meta-analysis of t-VNS in young healthy adults has found an overall moderate effect especially for improved cognitive performance especially executive function (Ridgewell et al., 2021). However the neuroanatomical substrates of persons with treatment-resistant depression or epilepsy are likely both widely variable, and grossly different to both a young cognitively healthy adult and a person with mild cognitive impairment (MCI) or dementia and dedicated larger studies are required to investigate if t-VNS has therapeutic potential in this population.

The purpose of this narrative review will be to outline the research to date investigating both cognitive outcomes of VNS in healthy and clinical populations, and the effect VNS has on HRV as a measure of autonomic tone. The mechanisms of action of VNS including neurotransmitter release, local increased cerebral blood flow and modulation of peripheral hemodynamics are discussed and future research recommendations outlined.

## Anatomy and Physiology of the Vagus Nerve

The longest nerve in the body, the vagus nerve derives its name from the Latin for ‘straying’ or ‘wandering.’ Aptly named, the nerve has an extensive course, traveling from the medulla to the gut. The vagus nerve’s function is to transmit information to and from the central nervous system (CNS) regarding control of the gastrointestinal, cardiovascular, and respiratory systems. It is comprised of approximately 80% afferent and 20% efferent fibers (Foley and Dubois, 1937; Agostini et al., 1957) including A, B and C fibers classified by conduction velocity (Erlanger and Gasser, 1937). Vagus neurons may involve visceral (cardiac, bronchopulmonary, gastrointestinal) or somatic (soft tissues, muscles of palate, pharynx) modulation. Afferent fibers are further sub classified as general visceral afferent, general somatic afferent, or special visceral afferent. Two efferent fiber types are recognized, namely special visceral efferent and general visceral efferent (see Table 1). Fibers connect centrally to four vagal nuclei; the nucleus of the solitary tract (NTS) and spinal trigeminal nucleus which contain vagal afferent fibers and the nucleus ambiguous and dorsal motor nucleus of the vagus (DMN) from where vagal efferent fibers leave (Rutecki, 1990; Berthoud and Neuhuber, 2000).

**TABLE 1:** The constituent fibers of the vagus nerve.

	FIBER				
	Aα	Aβ	Aδ	B	C
Fiber diameter	13–20 mm	6–12 mm	1–5 mm	1–5 mm	0.4–2 mm
Gross anatomical structure	Large	Large	Large	Small	Small
Main function afferent	Somatic touch pain temperature	Somatic touch	Visceral: pain stretch chemical, temperature	Visceral	Visceral: pain stretch chemical, temperature
Main function efferent	Muscle tone	Muscle preganglionic	preganglionic	preganglionic	preganglionic
Myelin	+	+	+	+	−
Threshold mA	0.02–0.2 mA	0.02–0.2 mA	0.02–0.2 mA	0.04–0.6 mA	0.3–6 mA
Conduction velocity ms	8–120 ms	35–75 ms	3–30 ms	3–15 ms	0.5–2 ms
Purported effect of VNS on EEG	Synchronization	Synchronization	Synchronization	Synchronization	Desynchronization

*Adapted from Groves et al. (2005).*

Afferent vagus fibers enter the medulla at the level of the olive, and terminate primarily in the NTS (Beckstead and Norgren, 1979; Kalia and Sullivan, 1982). Each vagus nerve (VN) synapses bilaterally in the NTS; so vagal afferent information is processed bilaterally in the CNS (Henry, 2002). Second order afferent fibers from the NTS project most densely to the parabrachial nucleus of the pons (PBN) with the NTS also projecting to noradrenergic (locus coeruleus) and serotonergic (raphe nuclei) neuromodulatory systems (Rutecki, 1990; Saper, 2000). From here vagal information is relayed to a number of mostly subcortical structures, including the hypothalamus, the central nucleus of the amygdala, the bed nucleus of the stria terminalis, and the intralaminar thalamic nucleus. Vagal afferent information is also sent to the anterior insular cortex which communicates with more rostral regions of the cortex (orbital and ventrolateral prefrontal cortex) and also indirectly with the medial prefrontal cortex (Öngür and Price, 2000; Saleem et al., 2008).

These central structures are part of the central autonomic network (CAN) which is thought to be the origin of autonomic, behavioral, cognitive, and endocrine responses, capable of modulating the functioning of the autonomic nervous system (ANS) *via* descending pathways projecting onto sympathetic pre-ganglionic neurons in the spinal cord and onto the DMN at the origin of vagal efferents (Benarroch, 1993). The central connections of the DMN are considerable, with afferent projections arising from sites including the NST, magnocellular paraventricular nuclei and several medullary nuclei (Roges et al., 1980; Hansen, 2019). Whilst a minority of efferent fibers connect centrally, most DMN fibers project to GI organs *via* parasympathetic ganglia located close to or in the walls of viscera. Further efferent fibers originate from the nucleus ambiguous (NA), a motor nucleus located in the reticular formation of the medulla which gives rise to preganglionic neurons innervating the heart and lungs (Llewellyn-Smith and Verberne, 2011) which exert a cardio-inhibitory effect mediated *via* the sinoatrial and atrioventricular ganglia (Massari et al., 1995; Gatti et al., 1996). The right vagus nerve mostly innervates the sinoatrial node (involved in the pacemaker function of the heart) whereas the left vagus is mostly thought to innervate the atrioventricular node (regulating the force of contraction of the cardiac myocytes with less influence over heart rate) however comprehensive human studies confirming this precise delineation are needed (Coote, 2013). The dorsal branchiomotor division of the NA is the site of origin of efferent fibers innervating striated muscle of the palate, pharynx, larynx and upper esophagus.

**FIGURE 1:**
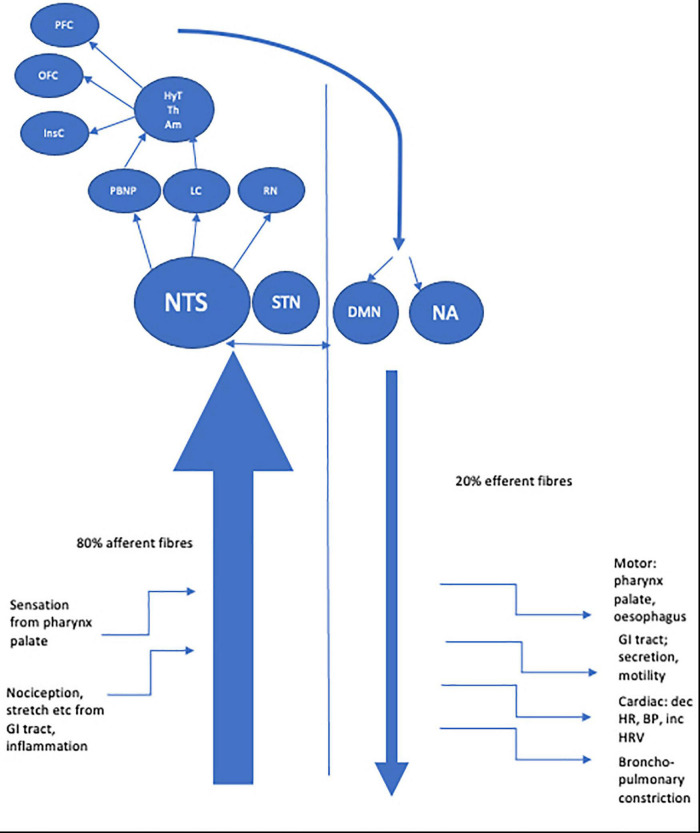
Schematic representation of afferent and efferent fibers of the vagus nerve and central projections.

See Figure 1 for a schematic representation of the VN fibers and central projections.

## History of Vagus Nerve Stimulation

Vagus nerve stimulation was initially proposed as a therapeutic intervention in 1871 (Neftel, 1871) and a device was designed to stimulate bilateral vagus nerves in the late 19^th^ century (Lanska, 2002). Preclinical studies in the 1930–1950s demonstrated *via* EEG signaling that VNS had cortical stimulating activity (Bailey and Bremer, 1938; Zanchetti et al., 1952), and could terminate canine seizures (Zabara, 1985, 1992).

Invasive VNS (iVNS) received United States regulatory approval for the adjunctive treatment of refractory seizures in 1997 and for use in treatment resistant depression in 2005 (O’Reardon et al., 2006). However, given the invasive nature of iVNS (requiring general anesthesia, thoracic implantation of a battery generator, and neck dissection to attach stimulating electrodes to the left cervical vagus nerve), the concept of non-invasive VNS was proposed in 2000 whereby, drawing on evidence from studies of auricular acupuncture, it was postulated that transcutaneous vagal stimulation could represent a valuable tool in epilepsy treatment (Ventureyra, 2000). Non-invasive VNS involves using stimulating electrodes on the skin to excite afferent vagal fibers and can be performed *via* the ear (transcutaneous auricular VNS: t-VNS) or the neck (transcutaneous cervical VNS: tcVNS). For the purposes of this narrative review non-invasive VNS will refer to auricular t-VNS.

**FIGURE 2:**
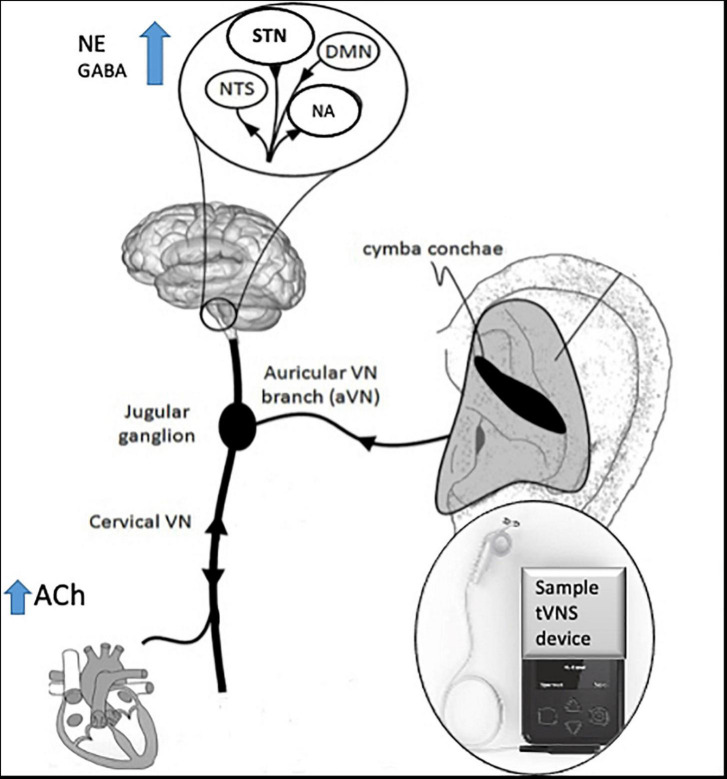
Schematic diagram of innervation of ABVN and central projections, adapted with permission from Kaniusas et al. (2019).

The technique of t-VNS exploits the peripheral anatomy of the vagus nerve, activating vagal afferent projections through stimulation of the auricular branch of vagus nerve (ABVN) at the ear (Peuker and Filler, 2002; Mercante et al., 2018) see Figure 2 for a schematic representation of the anatomy of the ABVN and central structures it modulates. Anti-seizure efficacy equivalent to iVNS was demonstrated in preclinical studies before the feasibility and therapeutic significance of this technique in humans were demonstrated (Stefan et al., 2012) and evidence from multiple functional brain imaging studies confirms significant activation of central vagal projections *via* this non-invasive method (Kraus et al., 2013; Frangos et al., 2015; Yakunina et al., 2017; Badran et al., 2018a).

Transcutaneous auricular vagus nerve stimulation waveforms can be delivered at a variety of different parameter settings which vary frequency (Hz), amplitude (mA), pulse width (μs-msec) and duration of stimulation. It is currently being investigated as a therapeutic intervention for a variety of medical disorders including epilepsy, migraine and cluster headaches, tinnitus, atrial fibrillation, Parkinson’s disease, schizophrenia, impaired glucose tolerance, obesity, and pain (Goadsby et al., 2014; Huang et al., 2014; Laqua et al., 2014; Hasan et al., 2015; Hyvarinen et al., 2015; Nesbitt et al., 2015; Stavrakis et al., 2015; Cakmak et al., 2017; Obst et al., 2020). There is particular interest in the evolving literature reporting the use of t-VNS in cognitive disorders (Broncel et al., 2020; Lam et al., 2021). Potential mechanisms of action include modulation of HRV, impacts on cerebral perfusion, and noradrenergic neuromodulation. The complementary role of vagus nerve stimulation in modulating neuro-cardiovascular and cognitive performance is explored in detail below.

See Figure 2 for a schematic diagram of the area of innervation of the ABVN and its central projections.

## Cognitive Performance and Vagus Nerve Stimulation

Brain imaging during t-VNS demonstrates strong activation of vagal projections to subcortical nuclei and frontal brain regions, i.e., superior frontal gyrus and medial frontal gyrus during stimulation (Kraus et al., 2013) (See below in “Mechanisms of Action” for further detailed discussion regarding the neuroanatomical structures modulated during VNS). Cognitive effects of both iVNS and t-VNS in both clinical populations and healthy volunteers will be examined under the following themes: Cognitive control, i.e., the non-automatic regulation of behavior to achieve a goal (Gonthier, 2014) a primarily executive function that involves suppression of goal-irrelevant stimuli *via* response and attention-inhibition (Tiego et al., 2018) and it primarily involves the lateral prefrontal cortex (Dixon, 2015); Language, both assessing categorical fluency a semantic memory language task involving the temporal lobe, and word recognition and retrieval which mostly involves episodic working memory, involving prefrontal cortex and medial temporal structures (Squire and Zola, 1998; Camina and Güell, 2017); Associative memory, a subcategory of declarative episodic memory and involves the ability to link disparate novel stimuli (Naveh-Benjamin, 2000); Emotion recognition as a subtype of cognition involves areas of the brain involved in perceiving social information including the medial prefrontal cortex and the orbitofrontal cortex (Bachmann et al., 2018) and regions implicated in emotional processing, including the cortical orbitofrontal cortex and the anterior cingulate cortex but also subcortical structures including the amygdala, hypothalamus, basal ganglia and the periaqueductal gray matter (van den Stock et al., 2011).

Interest in the potential role of VNS as a cognitive enhancer started following a preclinical rodent study of an inhibitory-avoidance task. Subjects received a single exposure to a foot shock followed immediately by VNS or sham. Those undergoing true VNS stimulation had longer step times demonstrating enhanced avoidance and this effect was modulated by the intensity of the stimulus, with 0.4 mA being an effective level of stimulation and 0.2 and 0.8 mA having no significant effect (Clark et al., 1995). Subsequent in-human trials tested word recognition in patients with intractable epilepsy who had iVNS devices implanted 2–24 weeks prior to testing. The stimulation parameters were 30Hz, 0.5 mA at 0.5 ms pulse width compared to an amplitude of 0.75–1 mA, and improved word recognition was only found in the group stimulated at the lower amplitude (Clark et al., 1999). These results paved the way for further investigation in this area as detailed below.

## Vagus Nerve Stimulation and Cognitive Control, i.e., Executive Function in Healthy Volunteers

Inhibitory control is commonly measured using performance on tasks such as the Stroop, Eriksen Flanker (Flanker), and Simon tasks, i.e., forced-choice reaction time tasks that require participants to selectively attend and respond to target stimuli whilst ignoring goal-irrelevant distracting stimuli (Kornblum et al., 1990; MacLeod, 1991; Eriksen, 1995).

Enhanced response times, as reflected by participants’ ability to stop a process and change to another response simultaneously and sequentially, and increased post error slowing were demonstrated during t-VNS (Sellaro et al., 2015; Steenbergen et al., 2015). Post error slowing refers to appropriate slowing after negative feedback or unforeseen errors and is linked to the activity of the locus coeruleus–norepinephrine (LC–NE) system and therefore postulated to be enhanced by VNS. As with the above trials, there were fewer false alarms during a more challenging paradigm with t-VNS when working memory processes were simultaneously engaged (Beste et al., 2016) and improved response selection and control performance was demonstrated with t-VNS in a serial reaction time test in young volunteers (Jongkees et al., 2018). In a sequence learning paradigm, the presentation of so-called reversal trials is associated with longer response latencies as compared to non-reversal trials, a result attributable to the ‘inhibition of return’ type phenomenon. Inhibition of return refers to an inhibitory after-effect of attention whereby, following exogenous orientation of attention to a stimulus, processing of stimuli at this location is first facilitated and then inhibited (Wang et al., 2018). Jongkees et al. (2018) demonstrated that active t-VNS, as compared to sham stimulation in the context of a serial reaction time test, reduced reaction time for reversal trials, eliminating the inhibition of return like effect described above.

In a similar experimental set up, increased attention, globally enhanced accuracy and reduced performance costs were demonstrated in a Stop-Change paradigm with t-VNS (Keute et al., 2020).

Results in this area have not been uniformly positive. In a testing paradigm in healthy volunteers using higher than average amplitude settings (see Table 2) there were no improvements in a Stroop test, Modified Flanker test or a number/letter working memory task with t-VNS. Improved accuracy in a dimensional change card sorting task was however noted (Borges et al., 2020). Similar previous studies failed to show improved behavioral performance with t-VNS (Fischer et al., 2018; Ventura-Bort et al., 2018) however non-performance parameters, namely a frontal EEG signal (P3 amplitude) thought to change with response inhibition and higher salivary amylase levels, were noted in the intervention group (Ventura-Bort et al., 2018). Further studies investigating EEG amplitudes affected by t-VNS and cognitive control paradigms included one involving an acoustic rather than visual oddball paradigm. In this context, t-VNS augmented the P3 amplitude, and with random noise stimulation with t-VNS reaction times were reduced (Rufener et al., 2018). There are myriad potential reasons for replication challenges in this newly expanding area of research and may include stimulation parameter differences including lack of pre-testing active stimulation.

**TABLE 2:** Cognition and VNS in healthy volunteer populations.

COGNITION AND VNS: Healthy volunteers								
		Stimulation Parameters						
Study	iVNS/tVNS	Hz	mA	Pulse width	Time	Population	Task	Outcome
Steenbergen et al., 2015	tVNS	25Hz	0.5mA	200–300 μs	30 s blocks	Healthy young adult volunteers *n* = 30	Stop change paradigm	Enhanced response selection and faster response times when two actions executed in succession
Sellaro et al., 2015	tVNS left outer auditory canal	25Hz	0.5 mA	200–300 μs	30 s blocks	Healthy young adult volunteers *n* = 40	Modified Flanker test	Increased post error slowing during active tVNS
Jacobs et al., 2015	tVNS left external acoustic meatus	8Hz	5.0 mA	200 μs	17 min	Healthy older adults avg age 60.5 *n* = 30	-Face name recognition task −15 word learning test -Digit span forward/backward -Verbal fluency test -Concept shifting task -Letter digit subtraction -Stroop color word test	Higher number of accurate “hits” during tVNS for face name recognition
Beste et al., 2016	tVNS left inner ear	25Hz	0.5 mA	200–300 μs	30 s on/30 s off	Healthy young volunteers *n* = 51	Inhibitory control (go-no-go task)	Fewer false alarms in the more challenging paradigm, i.e., when working memory processes also engaged
Colzato et al., 2017	tVNS Left outer auditory canal	25Hz	0.5 mA	200–300 μs	30 s on/30 s off	Healthy young volunteers *n* = 38	Emotion recognition Reading the mind in the Eyes test	Enhanced emotion recognition for easy (not challenging) items suggesting it promoted the ability to decode salient social cues
Fischer et al., 2018	tVNS	25Hz	Avg 1.3 mA (0.4–3.3)	200–300 μs	Continuous	Healthy adult volunteers *n* = 21	-Adapted response conflict Simon task -Novelty oddball task	No behavioral change noted Down-regulated N2 potential EEG reading
Ventura-Bort et al., 2018	tVNS Left cymba conchae	25 Hz	−1.3 mA (0.4–3.3) active −1.49 mA (0.6–4.8) sham	200–300 μs	28 min task 1 7 min task 2	Healthy young volunteers *n* = 21	-Novelty oddball task -number version of the Simon task	-No difference with tVNS with difficult targets or novel stimuli -Difference between tVNS and sham stimulation (P3 amplitude) in EEG parameters for easy targets associated with larger increase in sAA levels after tVNS
Rufener et al., 2018	tVNS Left cymba conchae	25Hz	0.5 mA	250 μs	30 s on/30 s off Started 90 min prior to task	Healthy young volunteers *n* = 20 avg age 24.8	-Acoustic oddball paradigm (respond as quickly as possible whenever a target tone was detected)	- tVNS increased EEG parameter P3 amplitude - Random noise stimulation reduced the reaction time
Maharjan et al., 2018	tVNS at left ear both anterior (cymba conchae) and posterior of ear	−80Hz −10Hz -No stim	10–15 mA	180 μs in square waveform	25–35 min lead in time	Healthy adult males *n* = 18	Two olfactory tests (odor threshold test (OTT) and supra-threshold test (STT)	High frequency (80Hz) VNS positively modulated olfactory performance in healthy participants and showed significant increase in NIRS recordings of the right hemispheric orbitofrontal cortex
Colzato et al., 2018	tVNS left concha *n* = 40 sham left earlobe *n* = 40	25Hz	0.5 mA	200–300 μs	15 min lead in time	Healthy young volunteers *n* = 80 (50 females, 30 males, mean age 20.96)	Convergent and divergent thinking tasks	-Fluency scores were significantly higher in the active tVNS group (able to generate more answers) -tVNS affected cognitive flexibility, i.e., participants could think of more different categories than sham
Jongkees et al., 2018	tVNS left medial acoustic meatus	25Hz	0.5 mA	200–300 μs	30 s blocks 15 min lead in time	Healthy young adult volunteers *n* = 40	Serial reaction time test	Enhanced response selection process and action control performance
Burger et al., 2019	tVNS Left cymba conchae	25Hz	0.5 mA	250 μs	30 s on/30 s off 10 min lead in time	Healthy young volunteers *n* = 61	Computerized fear conditioning, fear generalization, and fear extinction paradigm	No difference in physiological and declarative indices of fear between tVNS and sham conditions
Mertens et al., 2020	tVNS cymba conchae	25Hz	0.5 mA for 16 0.54–0.57 mA for rest	250 μs	30 s during consolidation	Healthy volunteers −*n* = 41 age avg 22.2 −*n* = 24 age avg 55.1	Word recognition task	No effect on verbal word memory
Giraudier et al., 2020	tVNS left cymba conchae (active) or left earlobe (sham)	25Hz	Active 1.48 mA ± 0.59 sham 1.31 mA ± 0.5	200–300 μs	30 s on/30 s off Stimulated for 23 min 5 min before 13 min during and 5 in after lexical decision task	Healthy volunteers −*n* = 60 −46 = female -avg age 23.45	Lexical decision task and recognition memory task of selected German words (either emotionally charged or neutral) Also – BP, HR and sAA	Overall no effect of tVNS on task performance or word recognition memory – however higher recollection based memory performance was observed during tVNS than sham
Borges et al., 2020	tVNS	25Hz	2.19 mA (±0.93)	200–300 μs	30 s on/30 s off 4 min lead in time	Healthy adult volunteers *n* = 35	-Modified Flanker test -Spatial Stroop task -Number/Letter task -Dimensional change card sorting task	Only the DCCS shows improvement with tVNS
Keute et al., 2020	tVNS left cymba conchae	25Hz	2.37 mA (±0.16)	200 μs	30 s on/30 s off 30 min lead in time	Healthy adult volunteers *n* = 22	Stop Change paradigm (go-no-go task)	Globally enhanced accuracy across conditions -Reduced the performance costs of go/change response conflicts -increased attention
Sun et al., 2021 Study 1	tVNS Left cymba conchae Sham: no stimulation	25Hz	Online 0.7 ± 0.36 mA Offline 0.69 ± 0.38 mA Sham 0.73 ± 0.27 mA	500 μs	30 s on and 30 s off 25 min pre task (offline) or 15 min during task (online)	Healthy young volunteer *n* = 46 (25 female, average age 20.39 ± 1.96)	Spatial stimuli task Four blocks with 72 experiment trials in each block	Offline (pre-task stim for 25 min) tVNS significantly increased hits in spatial 3-back task but not rejections or reaction times
Sun et al., 2021 Study 2	tVNS left cymba conchae sham; active stimulation of earlobe	25 Hz	Active: 0.74 mA ± 0.37 Sham: 0.84 mA ± 0.39	500 μs	30 s on and 30 s off Both 25 min stimulation	Healthy young volunteers *n* = 58 (24 female, average age 19.9 ± 1.49)	Spatial stimuli task Four blocks with 72 experiment trials in each block	Offline (pre- task stimulation for 25 min) tVNS improved hits but not correct rejections or reaction time of accurate trials in spatial WM performance
Kaan et al., 2021	tVNS Left tragus	25Hz	1–6 mA	250 μs		Healthy young volunteers *n* = 33 control *n* = 29 experiment	-Word retention: – non-rhyming, easily separable words -rhyming words	tVNS was associated with higher accuracy but only when the items are phonologically similar
McIntire et al., 2021	Cervical VNS *via* gammaCore device cVNS vs. sham (*n* = 20 both groups)	25Hz	Not available	Not available	2 min cycles	Healthy young military recruits *n* = 40 (M:F 33:7) avg age 28 ± 6 years	34 h of continuous sleep deprivation Air Force–Multi-Attribute Task Battery (AF-MATB); simultaneously monitor and respond to four separate cognitive process tasks: a visual system alert monitoring task, a visual–motor tracking task, an auditory communication monitoring task and a management task	cVNS significantly improved objective arousal and multitasking for as long as 24-h post-stimulation Subjective ratings of fatigue also improved

The most recent studies in this area have involved a spatial stimulation and response inhibition multitask, with notable improved results in accuracy with 25 min pre-assessment t-VNS stimulation (Sun et al., 2021) and improved objective attention, arousal and multitasking ability in sleep deprived military personnel (McIntire et al., 2021).

## Vagus Nerve Stimulation and Language in Healthy Volunteers

Fluency scores in healthy volunteers during a convergent and divergent thinking task were significantly higher during active t-VNS at the left conchae, and categorical flexibility (i.e., participants’ ability to think of more and varied categories of nouns) was also significantly improved (Colzato et al., 2018). However, an experimental design investigating the difference in effect of t-VNS on word recognition memory in young compared to older volunteers (average age 22.2 and 55.1) whereby t-VNS was delivered for 30 s during the consolidation phase of a word recognition memory task showed no improvement in accuracy scores for immediate recall or delayed recognition in both age groups (Mertens et al., 2020). Possible reasons for this may be that 30 s of t-VNS may be insufficient for a non-invasive device to effectively stimulate the vagal afferent pathway, that longer and more repetitive stimulation of the vagus nerve might be required to effectively modulate hippocampal processes *via* synaptic plasticity. A recent investigation of word retention, stimulating the left tragus with t-VNS at again similar parameters but wider amplitude found improved accuracy in word retention but only in items that rhymed, i.e., were phonologically similar (Kaan et al., 2021).

## Vagus Nerve Stimulation and Associative Memory in Healthy Volunteers

Transcutaneous auricular vagus nerve stimulation has been tested in a group of healthy older adults to determine the technique’s impact on performance in a face-name association task (Jacobs et al., 2015). VNS was employed in the encoding and consolidation phases of the task with active and sham stimulation compared in a randomized crossover design. Active t-VNS was demonstrated to increase the number of ‘hits’ on the memory task. Stimulation parameters employed differed somewhat from those seen in the broader literature concerning the impact of VNS on cognitive function. A stimulation intensity of 5.0 mA, a pulse width of 0.2 ms, and a frequency of 8Hz were utilized, citing previous functional and electrophysiological studies (Kraus et al., 2007; Polak et al., 2009). A stimulation lead in time of 17 min was also utilized, which has been theorized to be beneficial for targeted neuronal plasticity (Hays et al., 2013).

## Vagus Nerve Stimulation and Emotion Recognition in Healthy Volunteers

This ability to recognize different emotions in others was investigated and found to be enhanced by t-VNS at the left outer auditory canal in young healthy adults but only for objectively easy, not challenging, items *via* the Reading the Mind in the Eyes test (Colzato et al., 2017). Subsequent investigations of fear conditioning and extinction in young volunteers, after previous positive studies, found that t-VNS at the left cymba conchae did not infer any difference in physiological or declarative indices of fear or improve fear extinction (Burger et al., 2019). Further studies are needed in this area to elucidate if t-VNS has a specific beneficial effect, given its ability to modulate both cortical and subcortical structures.

See Table 2 for parameters settings and outcomes in trials of VNS in healthy volunteers.

## Vagus Nerve Stimulation and Cognition in Clinical Populations

In this section we highlight the studies to date investigating the cognitive effects of VNS on clinical populations, mostly with treatment-resistant depression or epilepsy. Many studies investigating the role of VNS in clinical populations has involved invasive VNS (iVNS). A further potential confounder is the impact some of these underlying pathologies have on cognition, the altered medial temporal anatomy especially in cases of epilepsy and the medications used to manage these conditions can also have deleterious effects on cognition.

## Vagus Nerve Stimulation and Cognitive Control, i.e., Executive Function in Clinical Populations

Vagus nerve stimulation has been shown experimentally to have mixed results when examining the subdomain of decision making, specifically on the Iowa Gambling Task (IGT). In one paradigm eleven patients with refractory epilepsy and iVNS devices completed a gambling task involving control and experimental trials with active VNS synchronized to stimulate in the latter. Whilst improved performance was demonstrated in the earlier part of the task, this trend was reversed later in the experimental trial with active stimulation trending toward being detrimental to performance (Martin et al., 2004). Technical failure and a cumulative stimulation-dose effect were amongst the potential explanations proposed by the authors to explain this phenomenon. Decision-making may depend on intact working memory (Bechara and Martin, 2004) and several studies have demonstrated working memory involvement in the IGT (Bagneux et al., 2013) which may have affected results in this study.

Working memory refers to a cognitive process that provides temporary storage and manipulation of the information necessary for complex cognitive tasks (Baddeley, 2010). Literature concerning the impact of acutely administered VNS on working memory is promising but limited to a small number of studies. In one experimental paradigm, twenty participants with poorly controlled epilepsy were required to perform a computer-based Executive-Reaction Time (Executive RT) Test, wherein ability to memorize and store the orientation of a triangle and indicate its position in response to a go signal were assessed whilst VNS was delivered in a cyclic fashion. Active iVNS stimulation was associated with fewer errors in the subtask relying on working memory (Sun et al., 2017).

The effect of active iVNS on response inhibition was also assessed by employing a classic stop-signal task in participants with refractory epilepsy (Schevernels et al., 2016). Quicker response inhibition has been demonstrated during active stimulation in patients who had previously shown a larger therapeutic effect of VNS. The beneficial effects of VNS on cognitive control may be maximally demonstrated in so-called ‘VNS responders’ (for the primary clinical indication) as demonstrated by patients with iVNS devices who undertook the Eriksen Flanker task during both VNS ‘on’ and ‘off’ stimulation. Only those deemed VNS responders (i.e., those whose seizure frequency had decreased by >50% post-device implantation) had demonstrable improved reaction times and reduced distractor interference during active stimulation (van Bochove et al., 2018). There is a subcategory of patients with refractory epilepsy who do not respond to iVNS therapy, i.e., do not have seizure reduction of 50%, and deemed “non-responders.” It is notable that a current output of 2.28 mA was utilized in the VNS “responder” group and it’s possible that, in keeping with previous studies examining optimal amplitude for stimulation, that the higher amplitudes employed exceeded that at which cognitive control is optimized for the iVNS “non-responders.” Further research is needed in this area in particular regarding stimulation parameters and iVNS responders.

## Vagus Nerve Stimulation and Language in Clinical Populations

In the first study of its kind, building on previous preclinical research, the impact of iVNS on word retrieval memory was assessed *via* an experimental protocol whereby participants with iVNS devices inserted for epilepsy control, were required to read a series of paragraphs, and subsequently identify words that were highlighted in the text. The study population comprised two groups of patients who were administered active (0.5–1.5 mA) or sham VNS, delivered 2-min after learning in the memory consolidation phase. An inverted U-shaped relationship was demonstrated regarding stimulus intensity and modulation of cognitive performance, with memory enhancing effects demonstrated only at moderate intensities, namely 0.5 mA (Clark et al., 1999). These results were in part corroborated by a subsequent study which employed higher stimulation intensities (>1.0 mA) and failed to demonstrate enhancement of verbal recognition memory, in fact demonstrating a reversible deterioration in figural memory (Helmstaedter et al., 2001). However, study design may have impacted cognitive outcomes here as delivery of stimulation was not restricted to the consolidation period. The propensity for iVNS to positively impact word retrieval memory in a population of patients being treated with iVNS for intractable epilepsy was highlighted again in 2006 whereby the impact of iVNS on performance in the Hopkins Verbal Learning Test was assessed, demonstrating a significant improvement in word retention when active (amplitude 0.5 mA) as opposed to sham stimulation was applied during memory consolidation (Ghacibeh et al., 2006b).

## Vagus Nerve Stimulation and Emotional Recognition in Clinical Populations

The effect of t-VNS on participants’ ability to recognize facial emotions in three experimental paradigms (graded presentation, static images and in a go-no-go task) was assessed in a group of adolescents diagnosed with major depressive disorder (MDD). In non-depressed controls t-VNS delivered at 1Hz, 0.5 mA 30 s block with 15 min lead in time, demonstrated enhanced recognition of emotions but notably led to a significant decrease in the ability of those with MDD to recognize sad emotions (Koenig et al., 2021).

See Table 3 for parameters settings and outcomes in trials of VNS in clinical populations and please see below “VNS, cognition and HRV” for a discussion of VNS in Alzheimer’s disease.

**TABLE 3:** VNS and cognition in clinical populations.

COGNITION AND VNS: Clinical Populations								
		Stimulation Parameters						
Study	iVNS/tVNS	Hz	mA	Pulse width	Time	Population	Task	Outcome
Clark et al., 1999	iVNS 2–24/52 post implantation	30Hz	−0.5 mA −0.75–1.5 mA	0.5 ms	30 s	Intractable epilepsy *n* = 10	Word recognition task	Improved word recognition memory only when 0.5 mA delivered post reading
Sjögren et al., 2002	iVNS Assessed at 3 and 6 months	20Hz	0.25 mA, increased 0.25 mA increments over 2 weeks then fixed	500 μs	30 s followed by 5 min pause	Probable Alzheimer’s *n* = 10 age 67 ± 7.6 8 women 2 men	Median change in ADAS-cog Median change in MMSE after 3 and 6/12 Depression, behavior and QOL variables	After 6/12 8 of 10 patients showed improvement from 3/12 ADAS-cog scores After 6/12 7 of 10 patients improved MMSE score by average 2.5 points No change in other variables
Martin et al., 2004	iVNS	30Hz	0.5 mA	500 μs	60 s	Intractable epilepsy *n* = 11	Iowa Gambling Task	Conflicting results, deleterious at higher doses
Merrill et al., 2006	iVNS At least 1 year of VNS treatment	20Hz	0.25 mA, increased in 0.25 mA increments over 2 weeks then fixed	500 μs	30 s followed by 5 min pause	Probable Alzheimer’s *n* = 17 (age 63 range 57–81) 11 women 6 men	Median change in ADAS-cog Median change in MMSE after 1 year Depression, behavior and QOL variables	At 1 year, 41% had improvement or no decline from baseline on ADAS-cog 70% had improvement or no decline on MMSE No change in other variables
Helmstaedter et al., 2001	iVNS 5–7/12 post implantation	30Hz	Mean 1.75 mA (range 1–2.5)	500 μs	30 s–4.5 min	Intractable Epilepsy *n* = 11	Word recognition task Design recognition task	Deterioration in figural recognition memory
Dodrill and Morris, 2001	iVNS 12–16/52 after implantation	30 Hz in high stim group 1 Hz in low stim group	Avg 1.3 mA in high simulation group Avg 1.2 mA in low stimulation group	500 μs 130 μs	30 s on every 5 min 30 s on every 3 h	Intractable Epilepsy *n* = 160	Wonderlic personell test, Stroop test, Digit cancelation, Symbol Digit Modalities	No significant changes were noted in the cognitive tests in low or high stimulation
Ghacibeh et al., 2006b	iVNS >3/12 post implantation	X	0.5 mA	x	30 s	Intractable Epilepsy *n* = 10	Hopkins verbal learning test	Improved retention index
McGlone et al., 2008	iVNS 12/12 post implantation	30 Hz	0.5–3 mA avg 1.72 ± 0.53	500 μs	30 s every 5 min	Intractable epilepsy *n* = 16	Memory Observation Questionnaire	Improved subjective and objective memory scores compared to baseline, but similar to medical management
Schevernels et al., 2016	iVNS >18/12 post implantation	Avg 25 (20–30)	Avg 2.3 mA (0.75–3.0)	Avg 431 μs (130–500 μs)	7 s on/ 18 s off	Intractable epilepsy *n* = 20	Stop signal task	VNS responders demonstrated quicker response inhibition
Sun et al., 2017	iVNS 2–130 months post implantation	30Hz	1.5–1.75 mA	250 μs	30 s on/48 s off	Intractable epilepsy *n* = 20	Executive reaction time test (go-no-go task)	Improved working memory (only when 3 participants with cognitive impairment removed)
van Bochove et al., 2018	iVNS	20 or 30 Hz	Avg 2.28 mA (0.75–3.0)	250 μs or 500 μs	7 s on/ 18 s off	Intractable epilepsy *n* = 17	Eriksen Flanker task	VNS responders demonstrated improved reaction times and decreased distraction interference
Koenig et al., 2021	tVNS Left conchae	1Hz	0.5 mA	250 μs	30 s on/30 s off 15 min lead in time	-Adolescents with major depressive disorder *n* = 33 control group: adolescents with headache *n* = 30	Facial emotional recognition in three tests 1. As a graded presentation 2. As static images 3. in a go – no -go task	-In non-depressed controls tVNS enhances the general ability to recognize emotions -tVNS specifically led to a decrease in the recognition of sad emotions in patients with MDD

## Linking Brain and Heart: Potential Mechanisms of Action of Vagus Nerve Stimulation-Mediated Cognitive Enhancement

There are many potential mechanisms through which VNS may exert its cognitive enhancing effects, including direct neurotransmitter release, increased cerebral perfusion to discreet neuroanatomical structures, reduced neuro-inflammation and *via* modulation of peripheral hemodynamics. For the purposes of this narrative review, we will analyze the link between cerebral blood flow, cerebral autoregulation and cardiac modulation. Beyond the scope of this review is how t-VNS may therapeutically affect the inflammatory cascade *via* activating the cholinergic anti-inflammatory pathway and the beneficial effects this may have in aging populations.

## Mechanism of Action: Vagus Nerve Stimulation and Local Neurotransmitter Release

The main neurotransmitters centrally released *via* the afferent projections of the vagus nerve are thought to be GABA and Norepinephrine (NE). For a comprehensive review of the preclinical and clinical studies detailing the evidence supporting the modulation of these neurotransmitters during iVNS and t-VNS see (Colzato and Beste, 2020).

As the primary inhibitory neurotransmitter in the brain, higher levels of GABA decrease cortical excitability, and is the accepted proposed method for VNS’ anti-seizure efficacy. It has been suggested that increased cortical inhibition due to high GABA levels can sharpen task-relevant representations in the cortex and inhibit competing responses, thereby facilitating response selection and inhibition processes (Munakata et al., 2011; de la Vega et al., 2014).

Norepinephrine is a crucial neurotransmitter modulating arousal and attention, and is primarily released *via* the locus coeruleus (LC). There are two distinct modes of LC firing that are associated with equally distinct modes of attentional strategy. Connections with the orbitofrontal cortex and anterior cingulate cortex are thought to drive the LC-NE system into one of these two stable states of activity, a high tonic (sustained) mode or a phasic (bursting) mode accompanied by moderate tonic activity (Aston-Jones and Cohen, 2005). This switching of attentional state *via* tonic LC activity is thought to result in a flexible attentional system that allows cycling between behaviors to find and meet task demands in one’s environment, i.e., the adaptive gain theory (Aston-Jones and Cohen, 2005).

Interestingly, and similar to the effects noted with iVNS stimulation levels and responses by Clark et al. (1995), moderate levels of NE augment prefrontal cortex function, whereas high and low concentrations of NE impair function, i.e., NE exhibits an inverted-U relationship between LC-NE activity and optimal performance on attention tasks (Berridge and Waterhouse, 2003). However, in general as NE levels rise executive function improves, likely *via* enhanced activation of the prefrontal cortex and frontoparietal control network (Xing et al., 2016; Unsworth and Robison, 2017). Inhibitory control for action cancelation is specifically enhanced with noradrenergic modulation, likely *via* this prefrontal cortical network (Chambers et al., 2009; Duann et al., 2009).

Older adults with more dense LC innervation (i.e., higher neuromelanin MRI contrast) had overall better performance on a reversal memory tasks (Hämmerer et al., 2018) and had improved cognitive reserve (Clewett et al., 2016). Similarly in a post-mortem study of patients with Alzheimer’s disease, lower LC cell integrity and greater cortical tangle density was associated with greater tau burden beyond the medial temporal lobes and worsening memory decline, identifying LC integrity as a promising indicator of initial AD-related processes (Jacobs et al., 2021).

Studies have also demonstrated a decline in GABA concentration in frontal and parietal regions in aging populations, areas crucial for cognitive control (Gao et al., 2013; Porges et al., 2017). NE and GABA may in fact work synergistically to facilitate executive functioning; GABA by encouraging response inhibition of task irrelevant stimuli and NE *via* the LC-NE system increasing frontal NE release and thus executive functioning (Ridgewell et al., 2021).

## Mechanism of Action: Vagus Nerve Stimulation Increases Cerebral Perfusion

Cerebral autoregulation is the phenomenon by which the brain receives the same cerebral blood flow (CBF) despite variations in perfusion pressure. The aim of autoregulation is to protect the brain against hypoxia and edema as a result of decreased or critically high arterial blood pressures respectively. Multiple factors physiologically modify autoregulation including blood CO2 levels, hypoxia etc. While still controversial, the ANS may play a prominent role in cerebral autoregulation in response to such stimuli, inducing vasodilation or constriction, and parasympathetic and sympathetic nerves are anatomically located in the same perineural sheath innervating cerebral arteries (Tamayo and Siepmann, 2021). The means by which VNS exerts its cognitive enhancing effect is probably multimodal, however modulating CBF is likely a crucial factor.

Multiple modalities have been utilized to assess for CBF changes due to vagus nerve stimulation, including position emission tomography (PET), functional magnetic resonance imaging (fMRI) and single photon emission computed tomography (SPECT) studies and trials of patients with iVNS treatment for epilepsy and depression have demonstrated a variety of CBF modulatory effects at specific cortical and subcortical areas. Increased CBF at the orbitofrontal cortex (Henry et al., 1998; Bohning et al., 2001; Lomarev et al., 2002; Mu et al., 2004; Vonck et al., 2008), temporal lobe (Ko et al., 1996; Lomarev et al., 2002; Liu et al., 2003; Vonck et al., 2008; Conway et al., 2012), insular cortex (Liu and Hu, 1988; Henry et al., 1998, 2004), bilateral frontal lobes (Sucholeiki et al., 2002), left dorsolateral prefrontal cortex (Kosel et al., 2011) and subcortical structures including thalamus, hypothalamus, basal ganglia and other nuclei (Narayanan et al., 2002; Sucholeiki et al., 2002; Conway et al., 2012) has been observed. For a comprehensive review see Chae et al. (2003).

Notably analysis undertaken during acute iVNS has noted bilateral decreased hippocampal CBF (Henry et al., 1998; Mu et al., 2004; Vonck et al., 2008). This has been replicated in t-VNS functional imaging studies which have confirmed stimulation and increased CBF at vagally innervated brain regions during auricular t-VNS and notably decreased perfusion at hippocampal regions (Kraus et al., 2007, 2013; Frangos and Komisaruk, 2017). T-VNS has also demonstrated efficacy in increasing arousal in comatose patients who respond to auditory signaling and again the brain regions noted on fMRI to be activated were similar to previous iVNS studies, including left superior temporal gyrus, left prefrontal cortex, left insular cortex, left middle frontal gyrus among other cortical and subcortical structures (Yu et al., 2021).

It is worth considering that intermittently stimulating neurons at different frequencies produces drastically different changes in neuronal behavior with low frequency stimulation inducing long term depression (LTD) and less connectivity while intermittent high frequency stimulation produces long term potentiation (LTP) and increased signaling (Lomarev et al., 2002; Kealy and Commins, 2010). Therefore acute VNS stimulates brain regions mostly involved in alertness and frontal processing, whereas chronic stimulation may improve LTP in classic memory-associated regions, including the hippocampus. Evidence for this can be seen in preclinical studies (Zuo et al., 2007) but also significant increases in hippocampal gray matter volume over time has been observed in patients with iVNS devices inserted for treatment-resistant depression (Perini et al., 2017). More recently, Near Infrared Spectroscopy (NIRS) has been utilized to monitor cerebral blood flow and increased frontal perfusion in patients with epilepsy was noted during iVNS when paired with a cognitive task (Kunii et al., 2021).

Both dementia and even its prodromal stage, MCI, are characterized by a reduction in cerebral blood flow (Mazza et al., 2011; Sierra-Marcos, 2017). A meta-analysis of twenty-six studies investigating CBF in MCI found overall reduced tissue oxygenation, CBF and velocity in MCI compared to healthy controls (Beishon et al., 2017) and studies are underway investigating the CBF changes that may occur with cognitive stimulation in MCI and dementia (Beishon et al., 2019). Similar findings have been noted in patients with Alzheimer’s disease, with reduced CBF in many cortical regions including temporal (Sandson et al., 1996; Alsop et al., 2000; Asllani et al., 2008; Yoshiura et al., 2009; Ding et al., 2014) parietal (Alsop et al., 2000; Johnson et al., 2005) and other regions including precuneus, frontal and posterior cingulate cortex (Alsop et al., 2008; Yoshiura et al., 2009).

## Mechanism of Action: Vagus Nerve Stimulation Modulates Peripheral Hemodynamics

As well as modulating central neurotransmitter release and cerebral blood flow, VNS has been shown to have positive peripheral modulatory effects in pathological states characterized by impaired autonomic regulation including postural orthostatic tachycardia syndrome (POTS) (Petelin Gadze et al., 2018) specifically patients with POTS and impaired vagal cardiac control, as defined by reduced HRV (Jacob et al., 2019). T-VNS has also shown benefits in modulating blood pressure in induced orthostatic hypotension (Tobaldini et al., 2019). These studies suggest VNS may have a role in positively manipulating the peripheral baroreceptor-reflex and thus cerebral autoregulation, and potentially may improve cortical perfusion *via* this route, however further dedicated studies are required to precisely delineate this relationship.

## Vagus Nerve Stimulation and Heart Rate Variability

Heart rate variability analysis can be performed *via* a variety of approaches and is based on the extrapolation of time intervals between each R wave peak (Shaffer and Ginsberg, 2017), discounting any ectopic beats or arrhythmias, e.g., atrial fibrillation. The most commonly applied methods to determine HRV are time-domain analysis and frequency/spectral analysis. Indices deriving from the time domain analysis quantify the amount of variance in the selected inter-beat interval employing statistical measures, such as the standard deviation of the normal beat intervals (SDNN) and the root mean square of successive differences between normal beats (RMSSD) (Shaffer et al., 2014). The spectral analysis of HRV identifies oscillatory rhythms that occur in specific frequency ranges. Three main components of the spectrums can be identified as: the very low frequency band (VLF), below 0.04 Hz, likely influenced by thermoregulatory mechanisms and circadian rhythms; the low-frequency band (LF) between 0.04 and 0.15 Hz in humans, a marker influenced by baroreflex (Furlan et al., 2019) sympathetic and parasympathetic modulation; the high-frequency band (HF) in the range from 0.15 to 0.4 Hz, a marker of vagal modulation that is influenced by respiratory activity (Montano et al., 2009; Shaffer et al., 2014). One of the limitations of HRV analysis is high within and between individual variability, which may be reduced by longer measurement intervals, i.e., 24 h but which is resultantly harder to process. For a comprehensive review on the various indices please see Merrick et al. (2017).

The ANS influences cardiac beat-to-beat interval length in response to several factors. The sympathetic and parasympathetic systems are the principal rapidly reacting systems that control heart rate. The two systems have different latency periods with sympathetic effects on heart rate slower than parasympathetic (Warner and Cox, 1962; Pickering and Davies, 1973; Koizumi et al., 1983) i.e., the parasympathetic system has the ability to alter heart rate within 1–2 beats, while sympathetic effects take up to 10 s to take effect.

Low HRV has been associated with poorer prognosis in cardiovascular diseases, cancer, Metabolic Syndrome and Alzheimer’s disease and it has been postulated that related pathophysiological mechanisms often contribute to their occurrence and progression, namely inflammatory responses, sympathetic overactivity, and oxidative stress (Entschladen et al., 2004; Thayer and Lane, 2007; de Couck et al., 2012). Lower vagal nerve activity has been found to be significantly correlated with oxidative stress (Tsutsumi et al., 2008), with inflammatory markers in healthy individuals as well as in those with cardiovascular diseases (Haensel et al., 2008) and anxiety disorders have also been characterized by low HRV (Chalmers et al., 2014). Experimental studies have long demonstrated the success of behavioral (Stein and Kleiger, 2003) and pharmacological (Sandrone et al., 1994) interventions in manipulating HRV. Increases in HRV seen with physical fitness training are associated with improvements in executive function (Hansen et al., 2004). The links between executive function and cardiac autonomic regulation were further highlighted by a recent study examining the impact of cognitive and motor training on HRV indices. Physical training alone failed to impact HRV in older adults whereas dual cognitive and motor training significantly improved global and parasympathetic autonomic nervous system activity (Eggenberger et al., 2020). These studies point toward a duality; the vagal communications between heart and mind can be bidirectionally manipulated to improve both parasympathetic control of HRV and, synergistically, executive cognitive function.

Preclinical research has noted that VNS, particularly to the right vagus nerve, increases vagally mediated (vm-) HRV measures (Huang et al., 2010; Sun et al., 2013). In a canine study, VNS treatment enhanced HRV at 4 and 8 weeks and reduced heart failure development (Zhang et al., 2009) and a Japanese study in rabbits founds that intermittent VNS, but not constant VNS, increased the HF (vagal) component of HRV (Iwao et al., 2000). Discrepancies in this preclinical work may be due to different species, devices and parameters but indicate that manipulating the vagus nerve electrically can have positive impacts on cardiac function and HRV.

## Vagus Nerve Stimulation and Heart Rate Variability in Healthy Volunteers

Transcutaneous auricular vagus nerve stimulation devices and their stimulation effect on HRV have been examined in several experimental paradigms involving multiple auricular positions, left vs. right ear stimulation, and different stimulation settings. There is a trend toward positive findings, i.e., improved HRV indices, with t-VNS in healthy volunteer populations when the right auricular branch of the vagus is stimulated (de Couck et al., 2017; Machetanz et al., 2021). It is notable that greater responses to t-VNS (i.e., improved vagally medicated HRV signals) have been demonstrated in those with higher sympathetic balance at baseline in both younger and older volunteers, both acutely and with 2 weeks t-VNS at home for 15 min daily (Clancy et al., 2014; Bretherton et al., 2019). An experimental design comparing left and right t-VNS at multiple stimulation targets found that SDNN and RMSSD both were most significantly improved when the right cymba conchae and fossa triangularis were stimulated (Machetanz et al., 2021).

When specific parameters of stimulation at the left tragus were sequentially analyzed, the settings that had the most significant impact on heart rate analysis in young volunteers were 500 μs at 10 Hz (Badran et al., 2018b). Studies investigating the effect of t-VNS and 70-degree tilt table testing on HRV at the left tragus found that the RSA measure of HRV (HF domain) was also significantly increased during an orthostatic maneuver (Lamb et al., 2017) and similarly stimulation at the left cymba conchae during 75-degree tilt found that responsivity, i.e., degree of change of heart rate and systolic blood pressure during t-VNS were significantly higher during orthostasis compared to control (Tobaldini et al., 2019).

Research in this area has not been consistent. Some initial findings indicated improved HRV measures with t-VNS to the left cymba conchae but ultimately no difference compared to sham and at multiple intensities (Borges et al., 2019). In an experimental crossover design employing a variety of amplitudes at the right cymba, there was no positive signal in affecting HRV measures (Gauthey et al., 2020) and similarly t-VNS to the right tragus during rest and autonomic nervous system testing, with appreciably different stimulation parameters to what was previously cited in the literature, also did not have any effect on HRV (Sinkovec et al., 2021). Inconsistent results are likely due to the use of different anatomical sites and stimulation parameters being utilized, some with “lead in” times and some without, and reporting on this area has been of variable quality, and recent international consensus has called for standardized reporting of this research (Farmer et al., 2021).

## Vagus Nerve Stimulation and Heart Rate Variability in Clinical Populations

Initial studies in clinical populations involved patients with iVNS devices inserted for control of refractory epilepsy. The earliest study demonstrated a reduction in LF:HF ratio and significantly higher HF power was noted in the higher stimulation group than lower stimulation (see Table 4; Kamath et al., 1992). These results were not however replicated in further studies of similar populations with comparable stimulation settings at timeframes ranging from minutes to 1 year of stimulation (Handforth et al., 1998; Setty et al., 1998; Galli et al., 2003; Ronkainen et al., 2006; Barone et al., 2007). A small study analyzing HRV in patients with iVNS devices implanted for management of treatment-resistant depression noted an increase in the RMSSD (increased vagal predominance) during stimulation compared to baseline and healthy controls (Sperling et al., 2010). It is notable that iVNS devices are for the most part inserted to activate the vagus *via* its left cervical branch, thereby appropriately reducing adverse cardiac effects but also not demonstrably influencing HRV measures in these populations.

**TABLE 4:** VNS and neurocardiovascular assessment.

Neurocardiovascular assessment AND VNS								
		VNS Stimulation Parameters						
Study	iVNS/tVNS/site specific	Hz	mA	Pulse width	Time	Analysis parameters	Population	Result
Kamath et al., 1992	iVNS for refractory epilepsy (left cervical vagus)	2 Hz 30 Hz	0.1 mA 1 mA	130 ms 500 ms	Not specified	Baseline 45 min ECG readings pre implantation and at 2/52 post implant	Refractory epilepsy *n* = 8 High stimulation and low stimulation groups avg age 34 ± 7.8 range 21–47	HiStim group: LF:HF ratio decreased from 2.5 ± 1.5 preimplant to 1.5 ± 0.49 (*P* < 0.02) with iVNS Significantly higher HF power in the HiStim compared to LoStim group
Setty et al., 1998	iVNS for refractory epilepsy (left cervical vagus) implanted for minimum 1/12	30 Hz	Max tolerated threshold	750 μs	30 s on 5 min off	Pre and post stimulation ECG (7 min baseline, 2.5 min of stimulation and a 7 min post-stimulation)	Refractory epilepsy *n* = 10 (avg age 28 range 14–46) 8 men	No significant effect noted on HRV variables
Handforth et al., 1998	iVNS for refractory epilepsy (left cervical vagus)	30Hz in high stimulation group I Hz in low stimulation group	Avg 1.3 mA in high simulation group Avg 1.2 mA in low stimulation group	500 μs 130 μs	30 s on every 5 min 30 s on every 3 h	Study mainly aimed at seizure reduction in two groups (high vs. low stimulation) in refractory epilepsy	Refractory epilepsy High stimulation group *n* = 95 age 32.1 ± 10.8 Low stimulation *n* = 103 age 34.2 ± 10.1	“Autonomic function assessments revealed no significant changes in Holter function measures; mean heart rate, mean lowest or highest heart rate, heart rate variability, occurrences of bradycardia”
Galli et al., 2003	iVNS for refractory epilepsy (left cervical vagus)	30 Hz	0.25 mA adjusted	500 μs	30 s on every 5 min	24-h analysis of RR variability at baseline (t0), 1 month (t1, short-term VNS) and 36 months after VNS initiation (t2, long-term VNS).	Refractory epilepsy *n* = 7 (4 men) age 47 ± 11.2 range 34–63 f	No significant changes in HRV variables, trend to increased HF at night-time
Ronkainen et al., 2006	iVNS for refractory epilepsy (left cervical vagus)	30 Hz	2.9 mA avg	500 ms	30 s on 5 min off	Pre and 1 year post implantation 24 h Holter HRV variables	Refractory epilepsy *n* = 14 (eight male and six female age 34.3 ± 9.3; 20–52) compared to matched controls	VNS had no significant effects on any HRV indices despite a significant reduction in seizure frequency
Barone et al., 2007	iVNS for refractory epilepsy (left cervical vagus)	30 Hz	0.75–1.75 mA	500 μs	30 s on, 5 s off	24 h ECG holter at baseline and after 3/12 implantation	Refractory epilepsy 8 patients (age 32 range 9–65 2 men)	No significant change in HRV parameters after 3/12 iVNS
Sperling et al., 2010	iVNS (left cervical vagus) for treatment resistant depression (post implantation 6–40 months)	15–30Hz	0.25–2.5 mA	500 μs	30 s on 5 min off	ECG testing at baseline, switched on and switched off conditions	Patients with major depressive disorder (ICD-10) *n* = 9 (51.6 years, 5 women, 4 men) Compared to age and sex matched controls	RMSSD increased significantly in switched on conditions during stimulation (30 s) in six patients compared to stimulation-free intervals and baseline
Clancy et al., 2014	tVNS on inner and outer surface of the tragus of the ear Sham – on tragus but disconnected Either active or sham tVNS	30Hz	10–50 mA	200 μs	Continuous 15 min stimulation	HRV frequency and spectral analysis Muscle sympathetic nerve activity (MSNA) recordings	Healthy volunteers *n* = 48 age 20–62 years old (M:F 1:1)	Significant decrease in LF/HF ratio during active tVNS Greater response to tVNS in those who had higher sympathetic predominance at baseline (higher LF/HF ratio)
de Couck et al., 2017 Study 1	tVNS cymba conchae left or right ear vs. sham (earlobe)	25Hz	0.7 mA average	250 μs	30 s on/30 s off 10 min	HRV frequency and spectral analysis	Healthy older volunteer *n* = 30 age 23–58	Right stimulation alone significantly increased SDNN compared to baseline
de Couck et al., 2017 Study 2	tVNS cymba conchae right ear	25Hz	1 mA average	250 μs	30 s on/30 s off 1 h	HRV frequency and spectral analysis	Healthy older volunteer *n* = 30 age range 30–65	SDNN significantly increased after 35 min and after 1 h specifically in female participants LF and LF/HF significantly increased after 35 min of stimulation
Antonino et al., 2017	tVNS active – tragus- inner and outer surface sham ear lobe Electrodes placed bilaterally (1) active tVNS (2) sham- olunteer placed on tragus –no current (3) olunteer placed on the earlobe current applied	30 Hz	45 ± 1 mA	200 μs	Continuous 15 min	HRV, BP variability, cBRS	Healthy young male olunteer *n* = 13 age = 23 ± 1	Active tVNS acutely improved spontaneous cBRS, olunteer LF/HF ratio and evoked slight decrease in HR Nil change with two sham conditions
Lamb et al., 2017	tVNS left tragus/auditory meatus or sham (no current)	20Hz	5.6 mA range 3–11.3 mA	100 μs	unavailable	Postural HRV *via* Tilt Table Test Startle Blink Paradigm	Military veterans with PTSD and mild TBI *n* = 12 or healthy control *n* = 10 age 30 ± 7	Significantly increased RSA (HF HRV) in tilt during tVNS Trend toward reduced reactivity (*via* electrodermal response monitoring) to startle
Badran et al., 2018b Study 1	tVNS to the inner side of the left tragus (anode in the ear canal, cathode on the surface of the tragus) of the left ear for 9 different stimulation rounds sham = left earlobe crossover design	1Hz 10 Hz 25 Hz	At 100 μs: tragus 9.28 ± 2.56 mA earlobe 6.5 ± 1.83 mA At 200 μs tragus 5.32 ± 1.60 mA earlobe 3.64 ± 1.26 mA At 500 μs tragus 3.0 ± 0.93 mA earlobe 1.97 ± 0.70 mA	100 μs 200 μs 500 μs	Stimulation period (60s) recovery period (180s)	Heart rate analysis	Healthy young adult olunteer *n* = 15 (M:F 1:1) age 26.5 ± 4.9	Active stimulation olunteer HR more than control stimulation on with these parameters: 500 μs at 25 Hz 500 μs at 10 Hz
Badran et al., 2018b Study 2	tVNS to the inner side of the left tragus (anode in the ear canal, cathode on the surface of the tragus) of the left ear for 10 stimulation rounds sham = left earlobe crossover design	10 Hz 25 Hz	tragus- 2.09 ± 0.97 mA earlobe 2.04 ± 0.82 mA	500 μs	Stimulation period (60s) recovery period (90s)	Heart rate analysis	Healthy young adult olunteer *n* = 20 (M:F 1:1)	The parameters 500 ms at 10 Hz alone induced a significant decrease in HR
Bretherton et al., 2019 Study 1	tVNS left tragus 1 week later sham (electrodes on tragus but no current)	30Hz	2–4 mA	200 μs	15 min	Baroreceptor sensitivity §	Healthy participants aged ≥55 years *n* = 14 Age 69.11 ± 1.52	Baseline LF/HF ratio power significantly predicted response to tVNS where higher resting LF/HF ratio was associated with greater olunteer during tVNS
Bretherton et al., 2019 Study 2	tVNS left tragus no sham	30 Hz	2–4 mA	200 μs	15 min	Baroreceptor sensitivity, HRV frequency and spectral analysis	Healthy participants aged ≥55 years *n* = 51 Age 65.20 ± 0.79	Total power, mean RR interval, Δ RR, SDRR were significantly affected during tVNS A higher LF/HF ratio predicted a greater decrease to tVNS
Bretherton et al., 2019 Study 3	tVNS left tragus daily at home for 15 min for 2 weeks	30Hz	2–4 mA	200 μs	15 min daily for 14 days	HRV frequency and spectral analysis	Healthy participants aged ≥55 years *n* = 29 Age 64.14 ± 0.89	RMSSD, pRR50, SD1 and nSD1, were significantly higher after 2 weeks tVNS
Tobaldini et al., 2019	tVNS left cymba conchae Cross-over design 2-day protocol, 1 day with tVNS and a control day, at least 24 h difference	25Hz	1–6 mA adjusted to sensory threshold	200 μs	10 min supine stimulator on (rest tVNS on), 15 min orthostatic position with tVNS on (tilt tVNS on)	(1) ECG (2) Respiration (3) Non-invasive beat-to-beat arterial blood pressure at rest and during a 75° tilt test	Healthy young olunteer *n* = 13 (5 males, 8 females) age 27 ± 4 years	Clinostasis: tVNS reduced HR, systolic BP variability and cardiac and peripheral sympathetic modulation Responsivity of HR and BP to orthostatic stress during tVNS was significantly higher when compared to control
Borges et al., 2019 Study 1	tVNS to left cymba conchae	25Hz	0.5, 1, and 1.5 mA	200–300 μs	30 s on/off cycling 10 min stimulation	RMSSD	Healthy young olunteer *n* = 61 (16 female) avg age 23.32	Increase in RMSSD during stimulation compared to the resting phases for all mA settings
Borges et al., 2019 Study 2	tVNS to left cymba conchae	25Hz	1 mA Compared to 1.78 mA ± 1.13	200–300 μs	30 s on/off cycling 10 min stimulation	RMSSD	Healthy young olunteer *n* = 62 (26 females avg age 24.77)	RMSSD values showed a significant overall increase during the stimulation phase none of the different stimulation conditions significantly differed from each other regarding RMSSD values
Borges et al., 2019 Study 3	tVNS to left cymba conchae vs. sham (earlobe)	25 Hz	Active 2.5 mA ± 0.93) Sham 2.76 mA ± 1.01	200–300 μs	30 s on/off cycling 10 min stimulation each	RMSSD	Healthy young volunteers *n* = 60 (31 females, age avg 23.62)	No difference between active and sham stimulation
Sclocco et al., 2019	tVNS (1) to cymba conchae no current (2) to cymba conchae active during exhalation (3) to cymba conchae active during inhalation (4) sham to earlobe	25 Hz	(1) 1.6 mA ± 2.3 (2) 1.7 mA ± 2.4 (3) 1.4 mA ± 1.1	450 ms pulse width duration of 1 s	32 min	-Instantaneous HF-HRV index -four 8-min duration fMRI scans (1) passive control (2) active stimulation exhalation (3) active stimulation inhalation (4) active control	Healthy adult participants *n* = 16 (9 female, age 27.0 ± 6.6)	Exhalation tVNS but not inhalation enhanced cardiovagal modulation, i.e., increased instantaneous HF hRV index Exhalation found significantly signal at MRI site of LC/NTS
Gauthey et al., 2020	tVNS Cymba Crossover design	5Hz 20Hz active 5Hz sham	1.5 ± 0. mA 1.2 ± 0. mA 5.5 ± 1. mA	0.2 ms	10 min stimulation 10 min washout	Muscle sympathetic nerve activity (MSNA) recorded by microneurography at rest, during apnoea and tVNS HRV power and spectral analysis	Healthy, young male volunteers *n* = 28 (age 27 ± 4)	Acute right cymba tVNS did not induce any effects on HRV nor MSNA variables when compared to active control
Machetanz et al., 2021	tVNS right *n* = 7 left *n* = 6 -cymba conchae -cavum conchae -outer tragus -inner tragus -crus helicis -fossa triangularis	25 Hz at a periodicity of 1 Hz	0.2–2 mA 0.096–0.769 mA 0.05–0.4 mA	100 μs 260 μs 260 μs	90 s (i.e., 3 s × 30 s) at each stimulation site 144 parameter combinations	HRV power and spectral analysis	Healthy adults *n* = 13 (age 24 ± 3, 8 female)	Significant differences between right- and left-sided stimulation for the SDNN and RMSSD analysis only (increasing with right ear stimulation) HRV increases were highest at cymba conchae and fossa triangularis, to a lesser extent to stimulation at the inner tragus
Sinkovec et al., 2021	tVNS to right tragus during rest (60 min) and autonomic nervous system testing (15 min) (Valsalva, wet cold face, etc.) sham = no stimulation, preceded stimulation	20Hz	Adjusted individually to barely perceptible <150 μA	1 ms rectangular pulse width	1 h resting tVNS vs. sham 15 min ANST vs. sham	Continuous cardiac measurements with impedance cardiography Non-invasive arterial BP monitor ECG for HRV analysis	Healthy male volunteers *n* = 15 (age 23 range 20–25)	Indices of LV contractility, LV output, and LV work significantly decreased SBP and TPR significantly increased No difference HRV or ANST parameters

Please see Table 4 for further analysis of the specific neurocardiovascular assessments, specific t-VNS parameters and outcomes measures in discreet populations in this area.

## Vagus Nerve Stimulation, Cognition and Heart Rate Variability

Heart rate variability can be conceptualized as a biomarker of parasympathetic modulation, and it is associated with a network of brain regions involved in autonomic nervous system regulation, known as the central autonomic network (Benarroch, 1993; Thayer et al., 2009). This network, which comprises prefrontal cortical (anterior cingulate, insula, orbitofrontal, and ventromedial cortices), limbic (central nucleus of the amygdala, hypothalamus), and brainstem regions, areas of the brain intimately involved in emotional regulation and executive functioning, leading to the proposal that vagally mediated HRV may index these aspects of prefrontal cortical function (Thayer and Lane, 2007; Thayer et al., 2009). Higher HRV has been linked to better cognitive function in healthy adults including healthy older individuals (Frewen et al., 2013; Grässler et al., 2020) and a meta-analysis found a positive overall correlation (*r* = 0.09) between vagally mediated HRV indices and emotional regulation processes (including executive functioning, emotion regulation, and effortful or self-control) in mostly healthy participants across a number of age groups (Holzman and Bridgett, 2017).

Autonomic system dysfunction is common in patients with MCI, with studies suggesting MCI participants are 5.6 times more likely than controls to have autonomic dysfunction, specifically on assessment of HRV and cardiac reflexes (Collins et al., 2012). A meta-analysis of MCI with dementia also found autonomic dysfunction, as defined by reduced HRV, was significantly associated with cognitive impairment (da Silva et al., 2017). Reduced HRV is associated with worse performance on tests of global cognitive function, more than cardiovascular risk factors (Zeki Al Hazzouri et al., 2014).

Recent meta-analyses of HRV in patients with neurodegenerative conditions including MCI, Alzheimer’s disease, Lewy Body dementia (DLB), vascular dementia, Parkinson’s disease and multiple sclerosis found a significant, moderate effect (*r* = 0.25) indicating that higher HRV was related to better cognitive and behavioral scores, which was not influenced by mean age or cognitive status (Liu et al., 2022). These results were mirrored in a similar recent meta-analysis of patients with dementia compared to healthy controls, which found significantly lower resting HRV for parasympathetic function and total variability in those with dementia. On subgroup analysis then most striking differences, i.e., worse HRV analysis was found in those with MCI or DLB (Cheng et al., 2022).

Heart rate variability and CBF are linked *via* vagal afferents, and a meta-analysis revealed that HRV was significantly associated with regional cerebral blood flow in the ventromedial prefrontal cortex (including anterior cingulate regions) and the amygdala (Thayer et al., 2012). In both younger and older adults scanned while at rest, higher HRV is associated with higher medial prefrontal cortex and amygdala functional connectivity (Sakaki et al., 2016). The Neurovisceral Integration Model holds that HRV, executive cognitive function, and prefrontal neural function are integrally associated (Thayer et al., 2009).

In an interesting Swedish clinical trial in 2002, iVNS devices were implanted in a small group of patients with likely Alzheimer’s Dementia (AD) as defined by the criteria of the National Institute of Neurological and Communicative Disorders and Stroke and the Alzheimer’s Disease and Related Disorders Association (NINCDS-ADRDA), with a view to assessing its impact on cognition *via* memory test scores. In the primary trial, 10 patients with average Mini Mental State Exam (MMSE) scores of 21 (range 16–24) had iVNS devices implanted and the median change in MMSE and Alzheimer’s Disease Assessment Scale-cognitive subscale (ADAS-cog) scores among a battery of tests was assessed at 3 and 6 months, with improvements in both assessments noted in the majority (6 out of 10) of cases (Sjögren et al., 2002). The follow up trial by the same research group involved 17 patients with likely AD, who had iVNS devices implanted and had outcomes measured and available at 1 year post implantation. At 1 year, 7 of 17 (41%) had improvement or no decline from baseline in ADAS-cog scores and 12 of 17 (70%) had improvement or no decline in MMSE scores. There was no change in noted in other outcomes including depressive symptoms (Merrill et al., 2006). There are a small number of trials registered investigating the therapeutic potential of t-VNS in older populations, both healthy and with cognitive impairment (for a recent review see (Vargas-Caballero et al., 2022)) however there are no known published studies to date investigating t-VNS in populations with dementia or MCI, and the associated effect on HRV.

## Summary

There is mounting evidence of the potential benefits of VNS in myriad disease states, with notable promise in the area of cognition. VNS shows promise as a neuromodulatory technique in cognitive decline and this may be *via* its ability to regulate both cardiac autonomic function and increase cerebral perfusion. Dementia is a multifactorial process and together with reduced cerebral perfusion is associated with neuroinflammation and altered synaptic plasticity, both of which may also be favorably modulated by VNS. It has been noted that perfusion to cortical and subcortical areas increases with VNS, specifically to areas that modulate executive function and attention, i.e., insular, orbitofrontal and prefrontal cortex. These areas are hypothesized by the neurovisceral integration model to be crucial areas in modulating the ANS (Thayer et al., 2009). Given that the LC-NE system is intimately involved in the therapeutic effects of VNS, and likely improves cognition *via* norepinephrine release and improved executive performance, it is notable that the earliest stages of pathological tau accumulation in Alzheimer’s disease are seen in the LC. Whether this small midbrain nucleus will prove to be pivotal in our understanding of how to modulate the vagus nerve and harness its benefits cognitively remains to be elucidated. VNS can now be delivered safely and non-invasively *via* t-VNS devices with equivalent neuromodulatory effects on brain imaging as invasive devices, which broadens its therapeutic applicability considerably, especially to an older population with cognitive complaints for whom device implantation may not be feasible. Globally, the need for effective therapies to both treat the cause and symptoms of cognitive decline are needed urgently as rates of dementia increase due to population expansion. Dedicated studies into the potential therapeutic effects of t-VNS in early cognitive decline and dementia are needed. Research to date has been limited by myriad issues, including studies on cognition in clinical populations with altered neuroanatomy, lack of standardization in device usage, parameter settings, frequency of use, duration of stimulation. Minimum reporting standards have recently been published to help ameliorate some of these issues. Further rigorous studies of the therapeutic benefit of VNS are required, especially in populations with autonomic instability and cognitive decline.

## Author Contributions

HD did most of the research, writing, and editing of the article. Significant contributions were made by each author, specifically TD with manuscript reading, editing, and direction, SC with direction RE psychological assessments and plasticity, CF with neurocardiovascular assessments, ANS testing. PM and SK assisted significantly with overall editorial support and guidance. All authors contributed to the article and approved the submitted version.

## Conflict of Interest

The authors declare that the research was conducted in the absence of any commercial or financial relationships that could be construed as a potential conflict of interest.

## Publisher’s Note

All claims expressed in this article are solely those of the authors and do not necessarily represent those of their affiliated organizations, or those of the publisher, the editors and the reviewers. Any product that may be evaluated in this article, or claim that may be made by its manufacturer, is not guaranteed or endorsed by the publisher.
